# Survey data for COVID-19 vaccine preference analysis in the United Arab Emirates

**DOI:** 10.1016/j.dib.2020.106446

**Published:** 2020-10-22

**Authors:** Riham Muqattash, Ibrahim Niankara, Rachidatou I. Traoret

**Affiliations:** a Department of Accounting, College of Business, Al Ain University, Abu Dhabi, UAE; b Department of Finance and Banking, College of Business, Al Ain University, Abu Dhabi, UAE; c Department of Economics, New Dawn University (Université Aube Nouvelle), Ouagadougou, Burkina Faso

**Keywords:** Covid-19 pandemic, Vaccine preference, Vaccine skepticism, Willingness to vaccinate, Willingness to pay

## Abstract

In response to the call for interdisciplinary research on the potential effects of the coronavirus pandemic [1], this article presents a novel data set on individuals’ COVID-19 vaccine preferences in the United Arab Emirates (UAE). The menu of our stated preference survey questionnaire is framed based on the World Health Organization's (WHO) SAGE working group on immunization developed matrix of vaccine determinants [2], which was itself informed by a systematic review of peer reviewed and grey literature, and by the expertise of the working group. Our survey was designed in a bilingual (Arabic and English) format, using Google Forms platform and delivered to respondents aged 18 years and older using the snowball sampling method between July 4th and August 4th 2020, gathering a total of 1109 responses. Study participants were recruited across all seven emirates of the UAE (see Fig. 1). As presented in the conceptual framework (see Fig. 2), the data set comprises (i) respondents socio-economic and demographic information, (ii) respondents willingness to spend time, and money to get the Covid-19 vaccine, and (iii) the vaccine determinants identified by the WHO's SAGE working group on immunization.

## Specifications Table

**Table:** 

Subject	Infectious Diseases Prevention
Specific subject area	Health Economics. Econometric models (Random Utility Model) applied to stated infectious diseases’ vaccine preference data to understand the determinants of COVID-19 vaccine decision.
Type of data	Table
How data were acquired	Through a Survey (see supplementary files for a copy of the survey questionnaire, along with the web link to its online access)
Data format	Analysed, CSV and R formatted Data frames
Parameters for data collection	The target population is the set of all adults (18 years and older) living in any of the seven emirates of the UAE. No other parameters were used for the data collection.
Description of data collection	Data collection was conducted through an online questionnaire, which was delivered through snowball sampling methods to individual respondents through email, WhatsApp, and Microsoft Teams.
Data source location	The data collection covered the whole of the UAE national territory, which is made of seven emirates (See figure 1).
Data accessibility	Repository name: Mendeley repository [22] Direct URL to data: https://data.mendeley.com/datasets/pysxmjpkr4/1

## Value of the Data

• The data will be useful for researchers who want to investigate the determinants and the extent of COVID-19 vaccine acceptance/hesitancy/skepticism in the UAE.
• The data will also assist with studies interested in addressing the direct (financial) and indirect (time) barriers to COVID-19 vaccine program effectiveness in the UAE.
• The data will further assist with studies seeking to identify the determinants of individuals’ adherence to COVID-19 preventive measures in the UAE.
• The data could also serve researchers interested in studying the socio-professional and familial consequences of the COVID-19 pandemic in the UAE.
• Researchers interested in the influence of media on individuals’ attitudes towards COVID-19 in the UAE, would also find this data very handy.
• Overall, the data framework presented could also assist researchers to replicate data collection in any other national setting to address any of the above mentioned questions, including cross-country comparative analyses.

## 1 Data Description

The recent emergence and global spread of the severe acute respiratory syndrome coronavirus 2 (SARS-CoV-2) pandemic, widely referred to as “COVID-19”, has posed significant threats to public health systems, and exacerbated national economic conditions worldwide [3,4]. Despite its significance for designing an effective vaccination program against the COVID-19 pandemic, to date no data manuscript addresses nor provides data for analyzing COVID-19 vaccine hesitancy (or preference more broadly) within a health/economic system.

On March 2012 however, the WHO's SAGE group on immunization developed a matrix of vaccine demand determinants, categorized into contextual, individual & group, and vaccine-specific [5]. The menu of survey questions used to collect our currently shared COVID-19 vaccine preference data is framed based on this matrix. The link to our online survey questionnaire, along with a PDF copy of the actual questionnaire, and the csv format of the analyzed responses to the questionnaire are all provided as supplementary files to this manuscript. Although applied to COVID-19 vaccine preference analysis in the UAE, our presented data framework [see figure (2)] is general combining three key research paradigms in the scientific literature: the technology acceptance model (TAM), the framework on vaccine skepticism, and random utility theory.

**Fig. 1:**
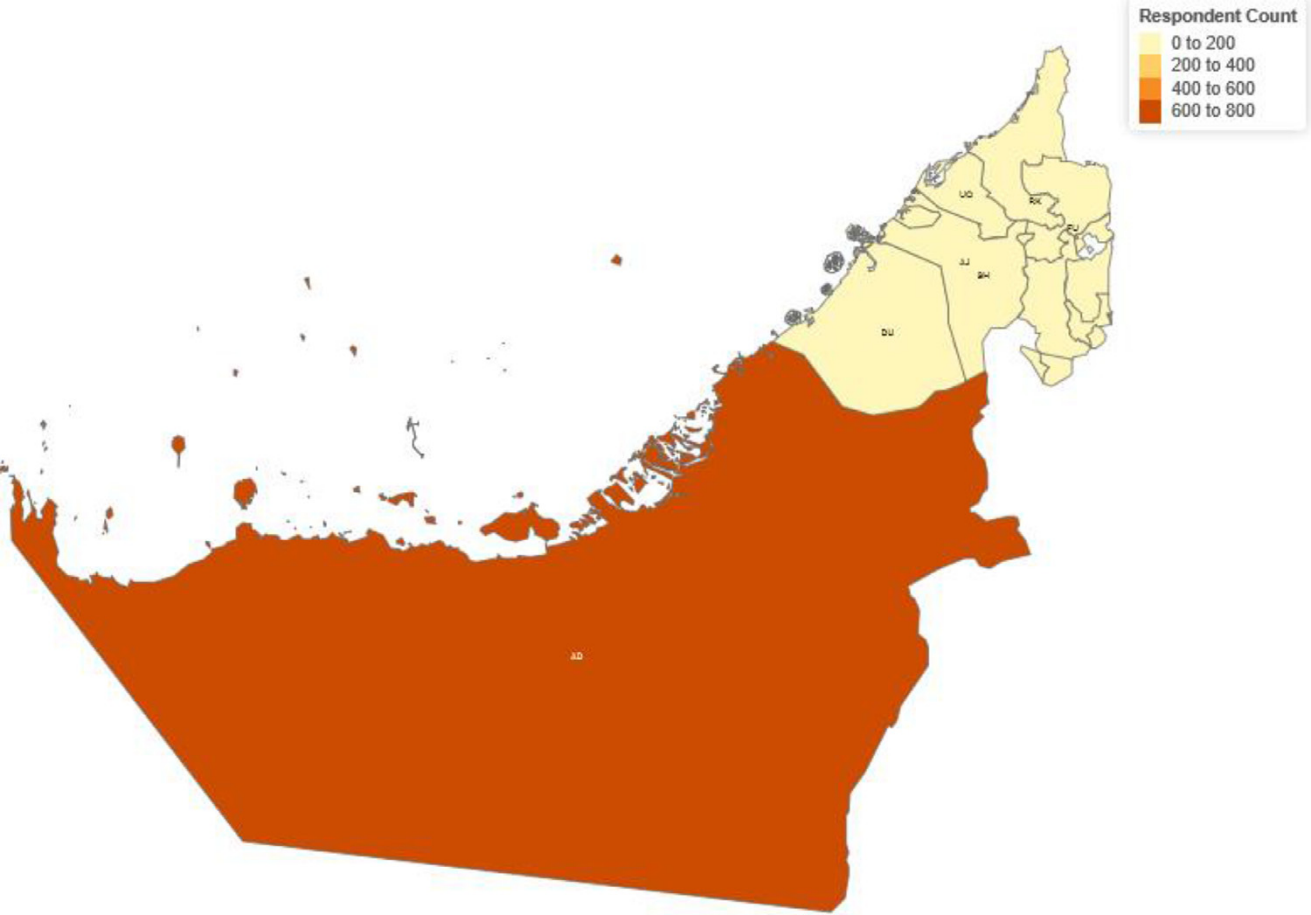
Spatial (geographical) coverage and individual count of the data collection. **Note**: Abu Dabi [AD: 796 (71.78%)]; Dubai [DU: 129 (11.63%)]; Sharjah [SH: 80 (7.21%)]; Ras Al Khaimah [RH: 13 (1.17%)]; Ajman [AJ: 50 (4.51%)]; Fujairah [FU: 34 (3.07%)]; Umm al Quwain [UQ: 7 (0.63%)].

Fig. 1 below shows the geographical map along with the frequency count, and relative percent frequency count of respondents across the seven emirates of the UAE. It can be noted that our data contains 1109 respondents, 796 (71.78%) of which are from Abu Dhabi, 129 (11.63%) from Dubai, 80 (7.21%) from Sharjah, 13 (1.17%) from Ras Al Khaimah, 50 (4.51%) from Ajman, 34 (3.07%) from Fujairah, and finally 7 (0.63%) from Umm al Quwain.

**Fig. 2:**
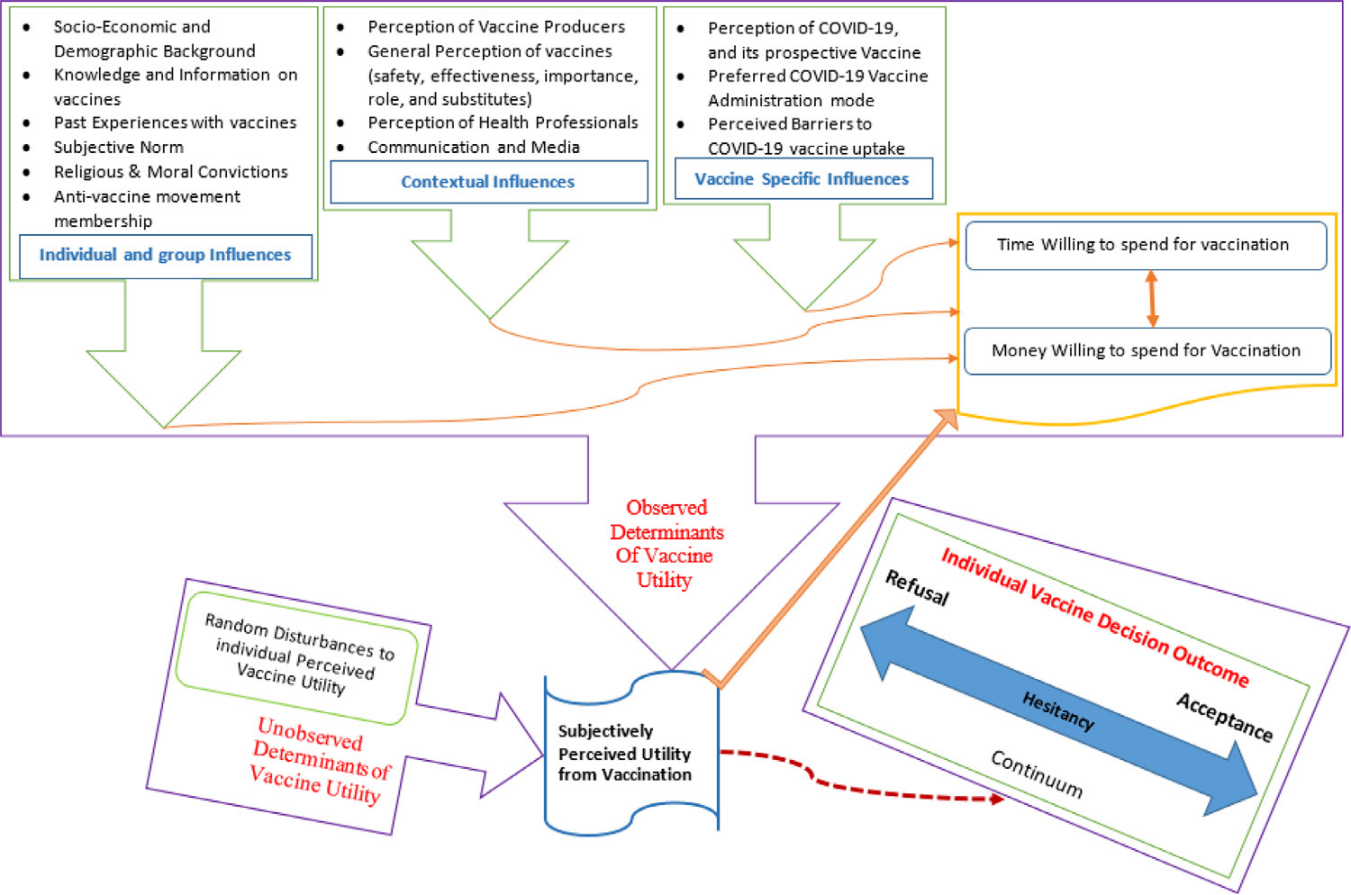
Random Utility Based Conceptual Framework for Individual's decision making about Covid-19 vaccination. **Note**: The framework combines the (bio) technology acceptance model with the vaccine skepticism framework, and Random Utility Theory. It suggests that observed determinants of vaccine utility combines with unobserved determinants to influence individual subjectively perceived utility from vaccination; this latter in turn identify the chosen position by the individual on the vaccine outcome continuum (Stated vaccine preference).

The recorded vaccine decision outcome are described in the Random Utility based conceptual framework in Fig. 2, which shows the relationships between the different collected variables. The framework suggests that observed determinants of vaccine utility combine with unobserved determinants to influence individual's subjectively perceived utility from vaccination; this latter in turn identify the chosen position by the individual on the vaccine outcome continuum (Stated vaccine preference).

In this representation however, given that the stated time each individual is willing to spend (indirect cost), and the amount of money the individual is willing to spend to get the vaccine (direct cost) are both under the control of the individual decision maker, they are necessarily endogenous determinants of the vaccine decision outcome. This implicitly means bidirectional relationships would prevail between these two determinants on the one hand, and the vaccine decision outcome on the other hand. Such bidirectional relationships (endogeneity) would have to be taken into account in any statistical modeling of the vaccine decision outcome.

The data collected based on this conceptual framework, through the online questionnaire include the socio-economic and demographic characteristics of the participants (see table 1 below); the personal and peer influences on individual perceived COVID-19 vaccine utility (see table 2 below); the contextual influences on individual perceived COVID-19 vaccine utility (see table 3 below); the vaccine specific influences on individual perceived COVID-19 vaccine utility (see table 4 below); and finally the vaccine decision outcome variable, along with the two endogenous vaccine decision determinants (see table 5 below).

## 2 Experimental Design, Materials and Methods

Our stated preferences data for a prospective COVID-19 vaccine in the UAE, was collected using a cross-sectional design. The survey was built using Google Forms platform and delivered to respondents using a snowball sampling strategy. The questionnaire was developed in bilingual (Arabic and English) format, and self-administered on a voluntary basis to participants across all seven emirates of the UAE (i.e. Abu Dhabi, Dubai, Sharjah, Ras Al Khaimah, Ajman, Fujairah, Umm al Quwain). In implementing our snowball sampling strategy, we (each co-investigator in the project) initially shared the web-based survey link through email on July 4^th^ 2020, to our primary contacts (aged 18 and above) living in the UAE, followed by a social media dispatch predominantly through WhatsApp and Microsoft Teams (MsTeams) channels. The primary respondents in our initial lunch of the survey were then requested to roll out the survey further after completion, by sharing the link with their own contacts in the UAE, while reminding their contacts to also share with their own after completion. The survey run for a month covering the period of July 4^th^ to August 4^th^ 2020, garnering responses from a total of 1109 participants. The responses were then downloaded from the Google forms platform, and imported into the R statistical software [6] for data treatment/preparation for statistical analysis.

Preliminary data treatments included the conversion of questions into coded variables that are readable by any standard statistical software. It also included the recoding of the levels of our qualitative (nominal and ordinal) variables with numbers, and the production of descriptive statistics and summary tables for study variables. The analyzed data has been made available in the Mendeley repository [7] in R format, while also provided as a supplementary material with this manuscript.

Table 1 below summarizes respondents’ socio-economic and demographic characteristics; Table 2 on the other hand depicts personal and peer influences on respondents’ perceived COVID-19 vaccine utility, while Table 3 conveys the contextual influences, and Table 4 describes vaccine specific influences on respondents’ perceived COVID-19 vaccine utility. Finally, Table 5 provides three key vaccine outcome variables: (i) the vaccine preference outcome (with varying degree of acceptances), (ii) the opportunity cost outcome (amount of time willing to spend for the vaccine), and (iii) the direct cost outcome (amount of money willing to spend for the vaccine).

**Table 1:** Socio-economic and demographic characteristics of the participants (*n*=1109).

variables	Description	Freq (n)	%
AGE	Respondent age category in years		
	1- [18 to 25]	143	12.89
	2- [26 to 35]	310	27.95
	3- [36 to 45]	437	39.40
	4- [45 and over[	219	19.75
Gender	Respondent gender		
	0- Male	309	27.86
	1- Female	800	72.14
MariStat	Marital status		
	1- Married	860	77.55
	2- Separated/divorced/Widowed	59	05.32
	3- Single	190	17.13
Nationality	Respondent nationality		
	0- Emirates	246	22.18
	1- Non-Emirates	863	77.82
ResidenCity	City of Residence (One of 7 as shown in Fig. 1)		
Education	Level of education		
	0- None	43	03.88
	1- High School	113	10.19
	2- Diploma	125	11.27
	3- Graduate	655	59.06
	4- Postgraduate	173	15.60
Occupation	Respondent sector of occupation		
	1- Not working	388	34.99
	2- Semi government	81	07.30
	3- Government	250	22.54
	4- Private	331	29.85
	5- Self-employed	59	05.32
IncomeMonthly	Monthly Income (1USD = 3.6725 AED)		
	0- None	149	13.44
	1- less than 10,000 EAD	344	31.02
	2- less than 20,000 EAD	275	24.80
	3- less than 30,000 EAD	184	16.59
	4- Above 30,000 EAD	157	14.16

**Table 2:** Personal and peer influences on individual perceived COVID-19 vaccine utility (*N* =1109).

variables	Description	Freq (n)	%
	**Knowledge and Information on vaccines**		
KnowVaccine	Can you tell me what a vaccine is?		
	0- No	478	43.10
	1- Yes	631	56.90
InfoSrcVaccns	Whom do you turn to for your information on vaccines?		
	Others	88	07.94
	Family OR relative	32	02.89
	A Friend	22	01.98
	A health worker	505	45.54
	The internet	462	41.66
EnouInfVacSafty	Do you feel you get enough information on vaccines and their safety?		
	0- No 1- Yes	650 459	58.61 41.39
	**Past Experiences with vaccines**		
EverNOTvaccin	Have you ever decided to not get a vaccination for yourself?		
	0- No 1- Yes	834 275	75.20 24.80
Any1BadReactVac	Do you know anyone who has had a bad reaction to a vaccine?		
	0- No 1- Yes	876 233	78.99 21.01
PastNegExpVacDiscrag	Do you remember any events in the past that would discourage you from getting the Covid-19 vaccine?		
	0- No 1- Yes	899 210	81.06 18.94
SatisfHlthProfAnsImu	How satisfied are you with your health professional/health worker's answers to your questions related to immunization?		
	0- Not at all 1- A little 2- A moderate amount 3- Quite a bit	105 177 490 337	09.47 15.96 44.18 30.39
	**Subjective Norm**		
ImportnCoVacEvery1	How important do you think it is for everyone to get the COVID-19 vaccine once available?		
	0- Not at all 1- A little 2- A moderate amount 3- Quite a bit	113 99 232 665	10.19 08.93 20.92 59.96
CoVaccCmplsry	Do you think COVID-19 vaccination should be compulsory or not, once available?		
	0- No 1- Yes	410 699	36.97 63.03
	Religious and Moral Convictions		
NoVaccRelgCult	Do you know anyone who does not take a vaccine because of religious or cultural reasons?		
	0- No 1- Yes	984 124	88.73 11.27
RiskngHlth	Do you think they are risking their health or the health of their family by not taking the vaccine?		
	0- No 1- Yes	384 724	34.63 65.37
ImpMenVaccWom	Do you think it is more important for men to get vaccinated than women?		
	0- No 1- Yes	954 155	86.02 13.98
	**Anti-vaccine movement Membership**		
AntiVaxxer	Do you identify as an anti-vaxxer?		
	0- No 1- Yes	900 209	81.15 18.85

**Table 3:** Contextual influences on individual perceived COVID-19 vaccine utility (*N* =1109).

Variables	Description	Freq (n)	%
	**Perception of Vaccine Producers**		
BeleiVacPrdcersIntrstHlth	Do you believe that vaccine producers are interested in your health?		
	0- No 1- Yes	371 738	33.45 66.55
TrustVaccProdSafeEffectVac	Do you trust vaccine producers to provide safe and effective vaccines?		
	0- No 1- Yes	410 699	36.97 63.03
	**General Perception of vaccines**		
PercVaccSaftyGenrl	How much do you think the following characteristics apply to vaccines in general? “Safe”		
	0- Not at all 1- A little 2- A moderate amount 3- Quite a bit	107 161 574 267	09.65 14.52 51.76 24.08
PercVaccEffGenrl	How much do you think the following characteristics apply to vaccines in general? “Effective”		
	0- Not at all 1- A little 2- A moderate amount 3- Quite a bit	109 166 532 302	09.83 14.97 47.97 27.23
PercVaccImportncGenrl	How much do you think the following characteristics apply to vaccines in general? “Important”		
	0- Not at all 1- A little 2- A moderate amount 3- Quite a bit	110 121 399 479	09.92 10.91 35.98 43.19
VacImunSysTrengh	Do you think vaccines strengthen the immune system?		
	0- No 1- Yes	327 782	29.49 70.51
AltrnPrevMesur	Do you believe that there are other (better) ways to prevent diseases which can currently be prevented by a vaccine?		
	0- No 1- Yes	417 682	37.60 62.40
	**Perception of Health Professionals**		
TurstVacAdvHlthProf	Do you trust the vaccine advice your health care provider gives you?		
	0- Not at all 1- A little 2- A moderate amount 3- Quite a bit	115 170 441 383	10.37 15.33 39.77 34.54
	**Communication and Media**		
InfoSrceCov	What is the most common information source you turn to, for information on COVID-19?		
	Others Government website News blogs News papers Radio Television The internet in general	32 373 53 42 11 114 484	02.89 33.63 04.78 03.79 00.99 10.28 43.64

**Table 4:** Vaccine specific influences on individual perceived COVID-19 vaccine utility (N =1109).

Variables	Description	Freq (n)	%
	**Perception of COVID-19, and its prospective Vaccine**		
SeriousCovDises	How serious do you believe the COVID-19 disease is?		
	0- Not at all 1- A little 2- A moderate amount 3- Quite a bit	40 84 336 649	03.61 07.57 30.30 58.52
ImportnCoVacc	How important do you believe the COVID-19 vaccine is?		
	0- Not at all 1- A little 2- A moderate amount 3- Quite a bit	106 88 244 671	09.56 07.94 22.00 60.50
ConcernCoVacc	How concerned are you about the COVID-19 vaccine?		
	0- Not at all 1- A little 2- A moderate amount 3- Quite a bit	100 160 394 455	09.02 14.43 35.53 41.03
	**Preferred COVID-19 Vaccine Administration mode**		
CoVaccPrefAdmnMod	What would be your preferred mode of administration, of the COVID-19 vaccine, once found?		
	None Orally Injected Nasal spray	239 310 488 72	21.55 27.95 44.00 06.49
	**Perceived Barriers to COVID-19 Vaccine uptake**		
FinCostCoVacPrevGet	Would the financial cost of the COVID-19 vaccine prevent you from getting it, if it was not provided for free?		
	0- No 1- Yes	532 577	47.97 52.03
TravelOver1HrCoVacc	If you have to spend more than one hour in travel time to get your COVID-19 vaccine, would you consider it important enough to travel for it?		
	0- No 1- Yes	366 743	33.00 67.00
TravelDiffEmirCoVacc	Will you be willing to travel to a different Emirate to get your COVID-19 vaccine, if it was not available in your emirate of residence?		
	0- No 1- Yes	403 706	36.34 63.66

**Table 5:** Vaccine outcome variables (N =1109).

Variables	Description	Freq (n)	%
WTGCoVacc	How willing are you to get the COVID-19 vaccine, once discovered?		
	0- Not at all 1- A little 2- A moderate amount 3- Quite a bit	279 229 356 245	25.16 20.65 32.10 22.09
MaxTimWillgSpndCoVacc	What is the maximum amount of time you would be willing to spend to get the COVID-19 vaccine, once discovered?		
	0- None 1- ]0 to 30 min[ 2- [30 to 60 min[ 3- [60 to 90 min[ 4- [90 to 120 min[ 5- [120 min and over[	63 512 95 57 242 140	05.68 46.17 08.57 05.14 21.82 12.62
MaxWTPCoVacc	What is the maximum amount of money (in dirham) you would be willing to pay for the COVID-19 vaccine, once discovered?		
	0- 0 AED 1- ]0 to 100 AED[ 2- [100 to 200 AED[ 3- [200 to 300 AED[ 4- [300 to 400 AED[ 5- [400 to 500 AED[ 6- [500 AED and over[	284 444 146 87 31 51 66	25.61 40.04 13.17 07.84 02.80 04.60 05.95

The first outcome represents the individual's willingness to get vaccinated, and is characterized by the individual's chosen position in the vaccine preference continuum, as indicated by the answer to the question “How willing are you to get the covid-19 vaccine, once discovered?”, with the alternatives defined as “vaccine refusal” if chosen option is (0-not at all); “vaccine hesitant” if chosen option is (1-a little; or 2-Moderate amount); “vaccine acceptant” if chosen option is (3- quite a bit).

As the stated opportunity cost of vaccination the second outcome variable captures the time the individual is willing to spend to get the vaccine, and is the answer to the question “What is the maximum amount of time (in minutes), that you would be willing to spend to get the covid-19 vaccine, once discovered?”, with 6 ordered outcomes (0- None; 1- less than 30 min; 2- 30 to 60 min; 3- 60 to 90 min; 4- 90 to 120 min; 5- over 120 min).

Finally, the stated direct financial cost of vaccination as the third outcome variable is the answer to the question “What is the maximum amount (in dirham), that you would be willing to pay for the covid-19 vaccine, once discovered?”. It has 7 potential choice options (0- 0 AED; 1- less than 100 AED; 2- 100 to 200 AED; 3- 200 to 300 AED; 4- 300 to 400 AED; 5- 400 to 500 AED; 6- over 500 AED), where it should be noted that a fixed exchange rate parity of 3.6725 AED/USD exists between the UAE dirham, and the U.S. dollar.

## Ethics Statement

Data collection was conducted according to the Declaration of Helsinki. Respondents’ participation was completely consensual, anonymous, and voluntary.

## Declaration of Competing Interest

The research project did not receive financial support from any institutions. The authors declare that they have no known competing financial interests or personal relationships that have, or could be perceived to have, influenced the work reported in this article.

## Appendix Supplementary materials

Image, application 1
