# Supplementary material for: Survey data for COVID-19 vaccine preference analysis in the United Arab Emirates
Source: Data Brief. 2020 Oct 22;33:106446. doi: 10.1016/j.dib.2020.106446 (PMC7577918; doi:10.1016/j.dib.2020.106446)
Supplement: Supplementary file 1 [file mmc1.zip › Supplementary Files/Online Survey Questionnaire Link.docx]

Online survey Link: <https://forms.gle/NsRehAzAyUQ7Pzrz6>
