# Supplementary material for: Survey data for COVID-19 vaccine preference analysis in the United Arab Emirates
Source: Data Brief. 2020 Oct 22;33:106446. doi: 10.1016/j.dib.2020.106446 (PMC7577918; doi:10.1016/j.dib.2020.106446)
Supplement: Supplementary file 1 [file mmc1.zip › Supplementary Files/Survey Questionnaire - Google Forms.pdf]

The Determinants of COVID-19 vaccine acceptance in the United Arab Emirates (UAE).

Dear Respected Respondent
Greetings

We are conducting a survey to measure the Determinants of COVID-19 vaccine acceptance in the United Arab Emirates (UAE).
Based on your personal opinion, please complete the following survey, your participation is highly important and mostly appreciated.
Results of the survey will be reported in general with no indication of the respondent's identity.

Best Regards
Dr. Ibrahim Niankara / Associate professor in Al-Ain University
Email : ibrahim.niankara@aau.ac.ae

Dr. Riham Muqattash/ Assistant Professor in Al-Ain University
Email: riham.muqattash@aau.ac.ae

Socio-demographic characteristic

1. Age

- 18 - 25 year
- 26 - 35 years
- 36-45 years
- above 45 years

2. Gender

- Male
- Female

3. Marital Status

- Married
- Single
- Separated/divorced/Widowed

4. Nationality

- Emirates
- Non-Emirates

5. City of Resident

- Abu Dhabi
- Dubai
- sharjah
- Ras Al khaimah
- Ajman
- Fujairah
- Umm al Quwain

6. Education
   - Postgraduate
   - Graduate
   - Diploma
   - High School
   - Other

7. The number of family members.

8. Occupation
   - Government
   - Private
   - Self-employed
   - Not working
   - Semi government

9. What is your monthly income in dirhams ?
   - less than 10,000 Dh.
   - between 10,000 and 20,000 Dh.
   - between 20,000 and 30,000 Dh.
   - More than 30,000 Dh.

Knowledge awareness

10. Have you heard about the Covid-19 Pandemic?

- Yes
- No

11. Can you tell me what a vaccine is?

- Yes
- No

12. Do you think a vaccine is needed for Covid-19?

- Yes
- No

Beliefs, attitudes about health and prevention

13. Do you believe that there are other (better) ways to prevent diseases which can currently be prevented by a vaccine?

- Yes
- No

14. Do you think vaccines strengthen the immune system?

- Yes
- No

15. Thinking about covid-19 prevention: how often do you do the following to protect yourself and others? “Social distancing”

- Not at all
- A little
- Moderate amount
- Quite a bit

16. Thinking about covid-19 prevention: how often do you do the following to protect yourself and others? “wearing masks”

- Not at all
- A little
- Moderate amount
- Quite a bit

Experience with vaccination and Covid-19

17. Have you ever decided to NOT get a vaccination for yourself?

- Yes
- No

18. Do you know anyone who has had a bad reaction to a vaccine?

- Yes
- No

19. Thinking about covid-19 effects on you: how much has it changed your "work/professional life"?

- Not at all
- A little
- Moderate amount
- Quite a bit

20. Thinking about covid-19 effects on you: how much has it changed "social life"?

- Not at all
- A little
- Moderate amount
- Quite a bit

21. Thinking about covid-19 effects on you: how much has it changed your “family life”?

- Not at all
- A little
- Moderate amount
- Quite a bit

Health system and providers’ trust and personal experience

22. How satisfied are you with your health professional/ health worker’s answers to your questions related to immunization?

- Not at all
- A little
- Moderate amount
- Quite a bit

23. How much do you trust the vaccine advice your health care provider gives you?

- Not at all
- A little
- Moderate amount
- Quite a bit

Risk/benefit (perceived, heuristic)

24. How important do you think the covid-19 vaccine is?

- Not at all
- A little
- Moderate amount
- Quite a bit

25. How serious do you believe the covid-19 diseases is?

- Not at all
- A little
- Moderate amount
- Quite a bit

26. How concerned are you about the covid-19 vaccine?

- Not at all
- A little
- Moderate amount
- Quite a bit

Immunization as a social norm vs. not needed/harmful

27. How important do you think it is for everyone to get the covid-19 vaccine for themselves and their families?

- Not at all
- A little
- Moderate amount
- Quite a bit

Communication and Media environment

28. What is the most common information source you turn to for information on Covid-19?

- Television
- Radio
- News papers
- News blogs
- Government website
- The internet in general

29. Whom do you turn to when you need more information on vaccines:

- Ask a friend what they think?
- Ask a health worker ?
- Ask a family or other relative ?
- Go to the internet?
- Others

30. Do you feel you get enough information about vaccines and their safety?

- Yes.
- No.

Historical influences

31. Do you remember any events in the past that would discourage you from getting the covidid-19 vaccine for yourself or your Family?

- Yes.
- No.

Religion/ culture/ gender/socioeconomic influences

32. Do you know anyone who does not take a vaccine because of religious or cultural reasons?

- Yes.
- No.

33. Do you think they are risking their health or the health of their family if they do not take a vaccine?

- Yes.
- No.

34. Do you think it is more important for men to get vaccinated than women?

- Yes.
- No.

Policy framework

35. Do you think Covid-19 vaccinations should be compulsory or not, once available?

- Yes.
- No.

Geographic barriers / opportunity costs/direct costs

36. If you have to spend more than one hour in travel time to get your covid-19 vaccine, would you consider it important enough to travel for it?

- Yes.
- No.

37. Will you be willing to travel to a different Emirate to get your Covid-19 vaccine if not available in your Emirate of residence?

- Yes.
- No.

38. What is the maximum amount of time you would be able or willing to spend to get the covid-19 vaccine for yourself or your family?

- Less than 30 minutes.
- 30 minutes to less than 1 hour.
- 1 h to less than 1 h:30 min.
- 1 h:30 min to less than 2 hours.
- Over 2 hours.

39. Would the financial cost of the Covid-19 vaccine prevent you from getting it, if it was not Provided for free?

- Yes
- No

40. Would you be willing to pay for the Covid-19 vaccine, once discovered?

- Not at all
- A little
- Moderate amount
- Quite a bit

41. What is the maximum amount (in dirham), that you would be willing to pay for the covid-19 vaccine, once discovered?

- 0 AED
- Less than 50 AED
- 50 to less than 100 AED
- 100 AED to less than 200 AED
- 200 AED to less than 300 AED
- 300 AED to less than 400 AED
- 400 AED to less than 500 AED
- Over 500 AED

Mode of administration

42. If a Covid-19 vaccine is found, what would be your preferred mode of administration?

- I prefer a vaccine that is injected
- I prefer a vaccine taken orally
- With a nasal spray
- None

Pharmaceutical industry influences

43. Do you believe that vaccine producers are interested in your health?

- Yes
- No

44. Do you trust vaccine producers to provide safe and effective vaccines?

- Yes
- No

VACCINE SKEPTICISM QUESTIONS

45. How much do you think the following characteristics apply to vaccines in general? "Safe"

- Not at all
- A little
- A moderate amount
- Quite a bit

46. How much do you think the following characteristics apply to vaccines in general? "Effective"

- Not at all
- A little
- A moderate amount
- Quite a bit

47. How much do you think the following characteristics apply to vaccines in general? "Important"

- Not at all
- A little
- A moderate amount
- Quite a bit

48. Would you be willing to get vaccinated against Covid-19, once a vaccine is available?

- YES
- NO
- I already got COVID-19, but if I hadn't, I would
- I already got COVID-19, but if I hadn't, I would not

49. Do you identify as an anti-vaxxer?

- Yes
- No

This content is neither created nor endorsed by Google.

Google Forms
